# Molecular and pathological insights into *Chlamydia pecorum*-associated sporadic bovine encephalomyelitis (SBE) in Western Australia

**DOI:** 10.1186/1746-6148-10-121

**Published:** 2014-05-29

**Authors:** Martina Jelocnik, David Forshaw, Jennifer Cotter, Danny Roberts, Peter Timms, Adam Polkinghorne

**Affiliations:** 1 Institute for Health and Biomedical Innovation, Queensland University of Technology, Kelvin Grove, Queensland 4059, Australia; 2 Department of Agriculture and Food Western Australia, Albany, Western Australia 6330, Australia; 3 Faculty of Science, Health, Education and Engineering, University of the Sunshine Coast, Sippy Downs 4556, Australia

**Keywords:** Sporadic bovine encephalomyelitis, *Chlamydia pecorum*, Cattle, MLSA, Histopathology, Immunohistochemistry

## Abstract

**Background:**

Despite its global recognition as a ruminant pathogen, cases of *Chlamydia pecorum* infection in Australian livestock are poorly documented. In this report, a *C. pecorum* specific Multi Locus Sequence Analysis scheme was used to characterise the *C. pecorum* strains implicated in two cases of sporadic bovine encephalomyelitis confirmed by necropsy, histopathology and immunohistochemistry. This report provides the first molecular evidence for the presence of mixed infections of *C. pecorum* strains in Australian cattle.

**Case presentation:**

Affected animals were two markedly depressed, dehydrated and blind calves, 12 and 16 weeks old. The calves were euthanized and necropsied. In one calf, a severe fibrinous polyserositis was noted with excess joint fluid in all joints whereas in the other, no significant lesions were seen. No gross abnormalities were noted in the brain of either calf. Histopathological lesions seen in both calves included: multifocal, severe, subacute meningoencephalitis with vasculitis, fibrinocellular thrombosis and malacia; diffuse, mild, acute interstitial pneumonia; and diffuse, subacute epicarditis, severe in the calf with gross serositis. Immunohistochemical labelling of chlamydial antigen in brain, spleen and lung from the two affected calves and brain from two archived cases, localised the antigen to the cytoplasm of endothelium, mesothelium and macrophages. *C. pecorum* specific qPCR, showed dissemination of the pathogen to multiple organs. Phylogenetic comparisons with other *C. pecorum* bovine strains from Australia, Europe and the USA revealed the presence of two genetically distinct sequence types (ST). The predominant ST detected in the brain, heart, lung and liver of both calves was identical to the *C. pecorum* ST previously described in cases of SBE. A second ST detected in an ileal tissue sample from one of the calves, clustered with previously typed faecal bovine isolates.

**Conclusion:**

This report provides the first data to suggest that identical *C. pecorum* STs may be associated with SBE in geographically separated countries and that these may be distinct from those found in the gastrointestinal tract. This report provides a platform for further investigations into SBE and for understanding the genetic relationships that exist between *C. pecorum* strains detected in association with other infectious diseases in livestock.

## Background

Cases of *Chlamydia pecorum* infection in Australian livestock are not well documented with only sporadic reports of chlamydiosis described by district veterinarians in agriculturally productive areas in Australia [1-3]. In cattle, *C. pecorum* is known to cause sporadic bovine encephalomyelitis (SBE), characterised by systemic infection involving the central nervous system. Cattle with SBE present with fever, followed by depression, limb stiffness, diarrhoea and staggering [2,4]. The disease is often fatal without rapid antimicrobial treatment in high doses [5]. *C. pecorum* can also cause other diseases in ruminants including infertility, vaginitis [6], enteritis [7], mastitis [8], pneumonia [9], conjunctivitis and arthritis [10]. The majority of *C. pecorum* infections in cattle appear to be subclinical and asymptomatic while exerting chronic pathological effects on the health of infected animals [10,11]. A common feature of these infections has been the consistent presence of *C. pecorum* in the gastrointestinal tract [5,10,12]. It has been suggested that the major route of transmission is the faecal-oral route, although infected animals also shed chlamydiae from genital [13], ocular and nasal sites [14].

The detection of *C. pecorum* from different anatomical sites in infected animals associated with a range of diseases other than SBE, has raised questions over the pathogenic potential of *C. pecorum* and the exact relationship between different strains and disease. Molecular markers have been used to discriminate between livestock *C. pecorum* strains with different disease presentations [15,16]. Yousef Mohamad et al. (2008), using Multi Virulence Locus Sequence Typing (MVLST) of *C. pecorum* strains isolated from a range of hosts and disease states, showed significant genetic diversity within these strains and some evidence of separation of livestock *C. pecorum* strains associated with morbidity from those isolated from the faeces of healthy cows. More recently, we evaluated the use of Multi Locus Sequence Analysis (MLSA) of *C. pecorum* house-keeping genes to understand the molecular epidemiology of *C. pecorum* infections in Australian livestock [15]. This analysis revealed (i) identical *C. pecorum* sequence types (STs) at ocular and/or urogenital anatomical sites and/or in association with diseases such as conjunctivitis, polyarthritis as well as no disease; and (ii) mixed different *C. pecorum* STs at different anatomical sites within the same animal. The latter observation highlighted the importance of sampling and molecular typing of *C. pecorum* from all potential infection sites within the same host to discriminate between strains with different pathogenic potential.

In the current report, we apply our MLSA scheme alongside pathological and immunohistochemical descriptions to characterise the relationship between *C. pecorum* types and disease in an outbreak of SBE in Western Australian (WA) cattle. We describe histopathological changes in internal organs of affected calves and demonstrate systemic dissemination and tissue invasion of a *C. pecorum* by immunohistochemistry and PCR. Using molecular typing we detected one *C. pecorum* genotype (denoted with ST 23), associated with these SBE cases from WA, as well as other SBE cases from Australia, England and the USA. Interestingly, MLSA also identified the presence of a second genetically distinct *C. pecorum* type in the gastrointestinal tract of calves affected by SBE.

## Case presentation

### Clinical history

Affected animals were two Murray Grey calves, 12 and 16 weeks old, at pasture on a property at Gairdner on the south coast of Western Australia. Six calves, aged 12 – 16 weeks, from a herd of 80 had died over the preceding two month period. The attending veterinarian was told that calves died in similar circumstances every year with animals becoming progressively duller over 3–4 days before dying. Calf WA/B31, a 12 week old Murray Grey heifer calf, was markedly depressed, unresponsive to external stimuli, blind and markedly dehydrated. Calf WA/B65, a 16 week old Murray Grey heifer calf, was markedly depressed with a stiff legged gait. Blood samples for serological and molecular analyses were taken from both calves and they were then euthanized with intravenous pentobarbitone. Both were necropsied and a range of tissues (brain, heart, liver, lung, skeletal muscle, small intestine, abomasum, mesenteric lymph node, kidney and forestomach) preserved in 10% buffered formalin for histopathology. From WA/B31, aerobic bacterial cultures were performed on a brain swab, joint swab, lung and liver. From WA/B65, aerobic bacterial cultures were performed on swabs of brain, joint, heart and abdomen. Impression smears were made of submitted fresh brain and lung of WA/B65 and fixed in acetone for immunofluorescence testing.

Twenty four swabs from the conjunctiva, vulva and rectum of 10 unaffected cohorts from the same herd were also collected for *C. pecorum* specific qPCR.

### Significant necropsy findings of euthanized calves

Calf WA/B31 - The liver of was slightly swollen and the lungs reddened and firm with approximately 5% of ventral lobes affected. Joints were not grossly swollen but examination of a carpus and stifle revealed excess clear fluid. No abnormalities were noted in the brain.

Calf WA/B65 - Massive opaque white fluid exudation into pleural, pericardial and peritoneal cavities was observed. Fibrin coated serosal surfaces and fibrin clumps were seen free in the fluid. The lungs appeared collapsed but no obvious consolidation was noted. There was excess clear joint fluid in the carpal joints examined. No abnormalities were noted in the brain.

### Significant histopathological changes

In both calves, most medium and small vessels throughout the brain had perivascular, mostly mononuclear cell cuffs up to four cells thick. Occasional vessels contained fibrinocellular thrombi and multifocal aggregates of polymorphonuclear neutrophils (PMNs) infiltrated the parenchyma, some well-preserved but others with advanced karryorhexis and karyolysis and multiple well circumscribed random foci of malacia were present. Rare basophilic intracytoplasmic inclusions were present in endothelium or mononuclear cells in inflammatory foci. There were extensive areas of glial cell proliferation surrounding these lesions and patchy gliosis throughout, most pronounced in the brain stem. There was a diffuse, mostly mononuclear, meningeal cell infiltrate most pronounced in the cerebellum where meningeal vessels had prominent lymphocytic perivascular cuffs with lesser numbers of macrophages and PMNs. The morphologic diagnosis was multifocal, severe, subacute, meningoencephalitis with vasculitis, fibrinocellular thrombosis and malacia.

#### Calf WA/B31

Heart, liver, lung, skeletal muscle, small intestine, abomasum, lymph node, kidney and forestomach were examined.

In the epicardium, there was diffuse, focally intense, infiltration of PMNs with lesser numbers of macrophages and lymphocytes accompanied by mild to moderate oedema. Activated mesothelial cells over inflamed areas were cuboidal with expanded cytoplasm and enlarged nuclei. The morphologic diagnosis was diffuse, focally severe, subacute epicarditis.

Areas of lung seen to be red and firm were atelectatic and there was diffuse congestion with increased mononuclear cells and smaller numbers of PMNs in alveolar septa. The morphologic diagnosis was mild acute interstitial pneumonia.

No significant lesions were detected in other organs.

#### Calf WA/B65

Liver, kidney, heart, spleen, lung, forestomach and brain were examined.

Pleural, epicardial and pericardial surfaces were heavily infiltrated by mixed inflammatory cells, predominantly PMNs and lymphoplasmacytic cells. On the serosal surfaces of abdominal organs, activated mesothelial cells proliferated with numerous cells sloughing into the overlying fibrinous exudate within which there was much streaming basophilic material (presumed DNA from degenerating cells) and infiltrating leukocytes. In places there was an intense mononuclear cell infiltrate below the fibrinous exudate. The morphologic diagnosis was severe diffuse subacute fibrinonecrotic pleuritis, epicarditis and pericarditis. No significant lesions were detected in other organs.

### Immunohistochemistry (IHC)

Selected sections of brain ileum, spleen and lung were labelled using a commercial mouse monoclonal protein A purified genus-specific epitope of the *Chlamydia* lipopolysaccharide antigen (Progen Biotechnik catalogue number ACI-C). Briefly, after deparaffinization of 5 micrometer thick sections in xylene and rehydration through graded ethanol to water, antigen retrieval was performed by a 5 minute enzyme digestion (Proteinase K, Dako S3004). After endogenous peroxidase activity was inhibited by incubation for 5–10 minutes with 3% hydrogen peroxide (Dako S 2023), slides were incubated at 4°C overnight with the primary antibody diluted 1:50 in the supplied antibody diluent. After rinsing in Tris Buffered Saline, specific labelling was developed using a proprietary system (Envision™ + Dual Link, single reagent HRP Rabbit/Mouse, Dako K 4063 or K4061). Following rinsing in purified water, sections were counterstained with haematoxylin. As well as the sections from cases WA/B65 and WA/B31, brain sections from two further cases were retrieved from the archives of the Department of Agriculture and Food, Western Australia (DAFWA) where a diagnosis of SBE had previously been made on the basis of histopathology and serological positivity by complement fixation.

Control sections of normal bovine brain and single sections from single archived cases of the common bovine neurological conditions; polioencephalomalacia, lead poisoning, listeriosis, salt poisoning, histophilosis and bacterial meningitis were also tested.

**Figure 1:**
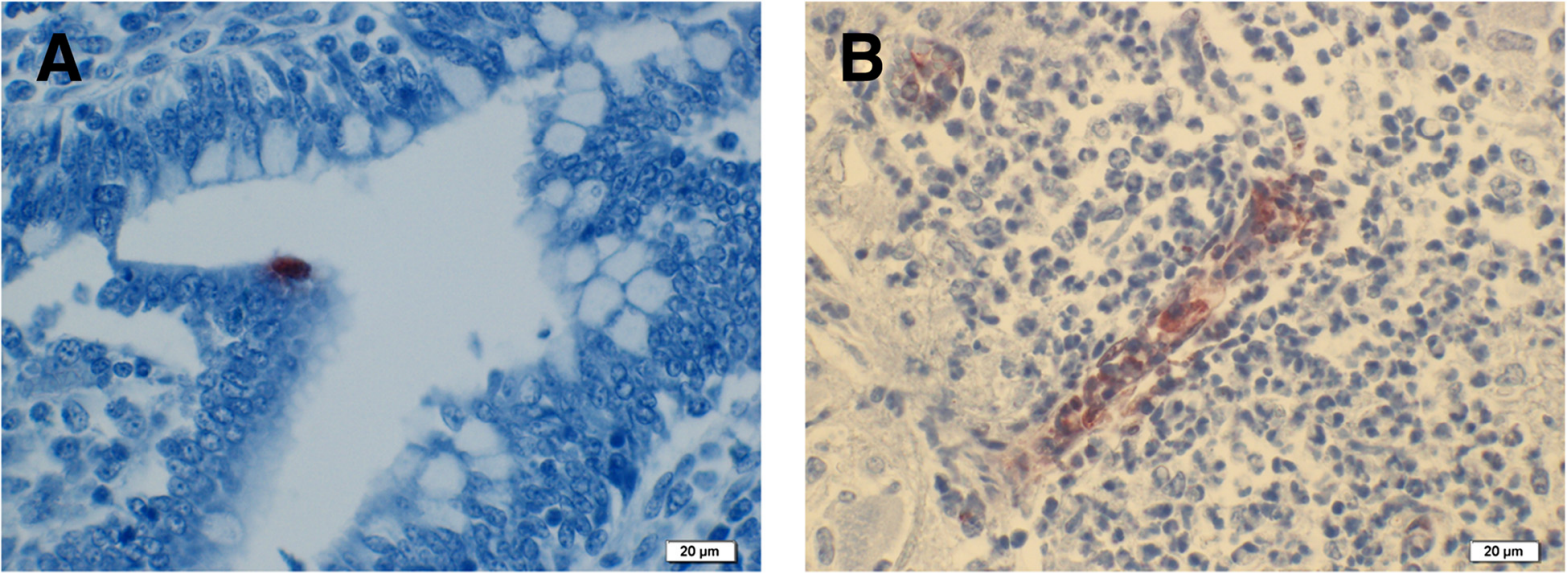
**Calf WA/B31 immunohistochemical staining using commercial monoclonal antibody to chlamydial LPS. A)** Ileum – Brown intracytoplasmic labelling of individual intestinal epithelial cells. **B)** Brain – Brown intracytoplasmic labelling of endothelium and intravascular mononuclear cells, in a focus of inflammation.

**Figure 2:**
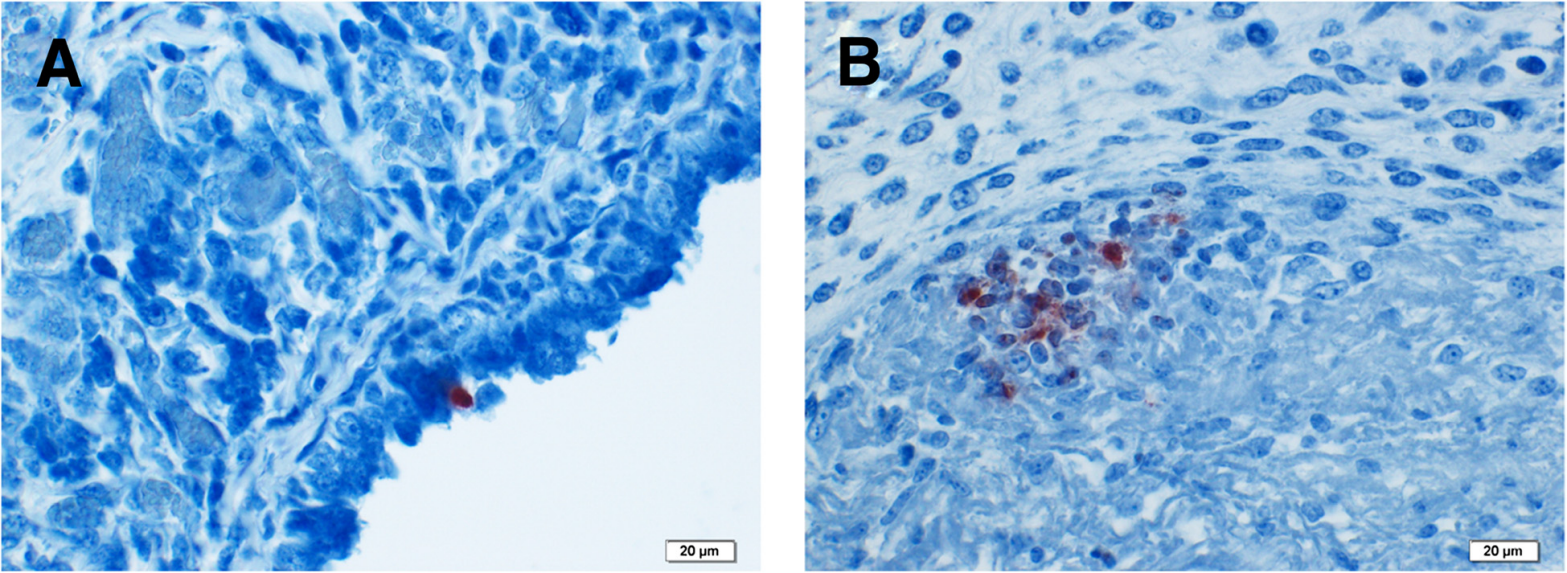
**Calf WA/B65 immunohistochemical staining using commercial monoclonal antibody to chlamydial LPS. A)** Spleen – Brown intracytoplasmic labelling of activated mesothelial cells. **B)** Lung – Brown intracytoplasmic labelling of mononuclear cells beneath layer of fibrin in overlying pleura.

Similar results were obtained upon examination of brain sections from calves WA/B31, WA/B65 and the two cases from the DAFWA archives.Calf WA/B31: There was intense but sporadic labelling of the cytoplasm of endothelial cells and intravascular mononuclear cells (Figures 1B) within inflammatory lesions and less intense speckled cytoplasmic staining of mononuclear cells in areas of malacia. There was rare intense labelling of single endothelial cells in blood vessels away from the foci of inflammation and in the meninges. In a single section of ileum from calf WA/B31, rare individual cells within the intestinal epithelium and endothelium of vessels in the lamina propria were labelled (Figure 1A).Calf WA/B65: Within the severe exudative pleuritis, there was labelling of mononuclear cell (macrophage or mesothelia) cytoplasm in a zone below the surface fibrin exudation in patchy but extensive zones (Figure 2B) and of the cytoplasm of individual mesothelial cells of the spleen (Figure 2A). No labelling was seen in the parenchyma of lung or spleen.

There was no labelling of control sections of normal bovine brain (data not shown) nor of single sections from archived cases of the common bovine neurological conditions as previously described.

### Other relevant test results

Immunocytochemistry – Acetone fixed impression smears of pleura and brain were stained with a fluorescein conjugated commercial monoclonal antibody to *Chlamydia* group specific lipopolysaccharide (Imagen, Oxoid catalogue no K610111-2) and examined with a fluorescent microscope. Punctate fluorescence was noted in the brain smear but not the lung smear.

Serology - Using the *Chlamydia* complement fixation test (CFT) which detects and measures antibodies (Ab) to the chlamydial genus specific lipopolysaccharide (LPS) antigen [17], antibodies were detected in serum from calf WA/B65 at a 1/64 dilution. Serum from calf WA/B31 was not tested.

Bacteriology - There were no significant bacterial isolates from brain swab, joint swab, lung and liver neither from calf WA/B31 nor from swabs of abdomen, pericardium, brain and a joint of calf WA/B65.

### *C. pecorum* species-specific quantitative PCR (qPCR) screening

In order to confirm the presence of *C. pecorum* in association with these cases of SBE, DNA was extracted using a QIAmp DNA kit (Qiagen, Doncaster, Australia) from lungs, livers, heart, brain, cerebrospinal fluid (CSF), kidney, mesenteric lymph node, ileal tissue and clotted blood of the two affected calves (B31 and B65) and from swabs collected from asymptomatic animals, as per manufacturer’s instructions. The testing of these swabs has been considered by the Queensland University of Technology (QUT) Animal Ethics Committee and approved under Tissue Use Notification #1100000362. This was used as a template for a previously described *C. pecorum*-specific qPCR, targeting a 202 bp region of the *C. pecorum* 16S ribosomal RNA (rRNA) [18]. The presence of *C. pecorum* DNA and the relative infectious loads in each tissue sample analysed are described in Table 1. With the exception of one sample (CSF in WA/Bovine 31), *C. pecorum* was detected in all tissues from both animals, with infectious loads ranging from very low as observed in kidneys and lymph node, to high as observed in brain, heart and lungs (Table 1). Nine of the 10 cohort animals with no apparent disease had < 15 *C. pecorum* 16S rDNA copies/μl of template, with *C. pecorum* DNA detected in six animals at ocular and vulval sites and three animals at one site only (ocular or vulval) (data not shown). The highest *C. pecorum* DNA positivity (70%), albeit at low infectious loads, was detected in vulval swabs, while no *C. pecorum* DNA was detected in the four available rectal swabs.

**Table 1:** ***C. pecorum *** **screening results for samples taken from the two SBE diseased calves**

**Host**	**Anatomical site**	***C. pecorum *** **qPCR screen**	***C. pecorum *** infectious load*
**WA/B31**	Lung A	POS	1.7x10^4^
	Lung B	POS	1.5x10^4^
	Liver**	POS	1.1x10^5^
	CSF	NEG	Nil
	Ileal tissue**	POS	1.8x10^3^
	Mesenteric lymph node**	POS	6.3x10^1^
**WA/B65**	Lung	POS	2.6x10^4^
	Liver**	POS	3.2x10^3^
	Brain	POS	4.0x10^5^
	Heart**	POS	9.7x10^3^
	Blood Clot	POS	7.2x10^2^
	Kidney**	POS	1.8x10^1^

*Mean No. of copies of 202 bp *C. pecorum* 16S ribosomal DNA PCR product per microliter of the tissue extracted DNA; ** Tissues in which no significant lesions were identified.

### *C. pecorum* MLSA from SBE field cases

To assess the phylogenetic relationships among *C. pecorum* strains detected in the two calves analysed in this study, we analysed concatenated sequence sets against the available HK gene sequences from seven other bovine *C. pecorum* isolates described worldwide and in association with several different aetiologies, utilising Bayesian methods. PCR amplification was successful for all samples except the WA/B31 lymph node and the WA/B65 clot and kidney samples.

The complete list of bovine *C. pecorum* isolates whose sequences were analysed in this study is described in (Table 2). In addition to the Australian samples from calves screened and typed in this study, HK gene fragment sequences from cases of SBE from the USA and England and from cases of pneumonia, arthritis, conjunctivitis and healthy faecal *C. pecorum* isolates were obtained from the *Chlamydiales* MLST website http://pubmlst.org/chlamydiales/[19], while the HK gene fragments sequences for the Italian bovine metritis PV3056/3 isolate were obtained from Genbank (accession number CP004033.1). Fragments of seven HK genes (*eno*A, *opp*A_3, gid*A*, *hem*N, *hfl*X, *fum*C and *gat*A) and *C. pecorum* specific primers to amplify them were previously used for typing Australian koala and livestock *C. pecorum* isolates. The PCRs and DNA sequencing of each *C. pecorum* HK gene was performed as previously described [15]. HK gene sequences of the bovine *C. pecorum* isolates described here are available in Genbank (KF714242 – KF714290). Additionally, we have identified allele numbers and STs for the Australian *C. pecorum* from the two calves, analysed in this study at http://pubmlst.org/chlamydiales/[19] (Additional file 1: Table S2).

**Table 2:** ***C. pecorum *** **bovine isolates used in the study**

**Isolate ID.**	**Country**	**Pathology**	**Isolate reference**
66P130	USA	Healthy (faeces)	[20]
BE53	England	Healthy (faeces)	[16]
E58	USA	Encephalomyelitis	[4]
FC-Stra	USA	Conjunctivitis	[21]
L14	USA	Pneumonia	[21]
LW623	USA	Arthritis	[21]
PV3056/3	Italy	Metritis	[22]
NSW/Bov/SBE	AUS	Encephalomyelitis	[15]
SBE	England	Encephalomyelitis	[16]
WA/B31/Ileal	AUS	SBE	This work
WA/B31/Liver	AUS	SBE	This work
WA/B31/Lung	AUS	SBE	This work
WA/B65/Brain	AUS	SBE	This work
WA/B65/Heart	AUS	SBE	This work
WA/B65/Liver	AUS	SBE	This work
WA/B65/Lung	AUS	SBE	This work

Chromatograms and sequences of the seven HK gene fragments were analysed using the Geneious Pro 6.0.4 software package [23]**,** as previously described [15]. Sequences of individual genes and concatenated gene sets were aligned using ClustalW [24]. The level of individual HK fragments as well as the concatenated sequences polymorphisms were determined by analysing the number of synonymous (d_s_) and non-synonymous (d_n_) substitutions per site, Jukes-Cantor corrected, and the number of segregating sites (Δnt), haplotypes and haplotype diversity (H) using DnaSP 5.0 (Additional file 2: Table S1) [25]. As observed in Additional file 2: Table S1, HK gene fragments and/or concatenated sequences are both under negative evolutionary selection with low d_n_/d_s_ ratios ranging from 0.0 – 0.15, with the number of synonymous substitutions per synonymous site (d_s_) occurring 3.7 times more than non-synonymous substitutions per non-synonymous site (d_n_). As such, sequences of the HK genes, being representative of overall bacterial chromosomal change, can provide effective phylogenetic resolution of bacterial strains [26].

Best-fit models of nucleotide substitution for constructing phylogenies of our data sets were estimated by considering eleven substitution models using jModelTest v.2.1.1 [27]. A mid-point phylogenetic tree containing all 16 bovine *C. pecorum* strains was constructed based on concatenated MLSA sequences, using the program MrBayes [28] with the HKY85 + I + G model, as implemented in Geneious Pro 6.0.4. Run parameters included four Markov Chain Monte Carlo (MCMC) chains with a million generations, sampled every 100 generations and with the first 1000 trees were discarded as burn-in.

**Figure 3:**
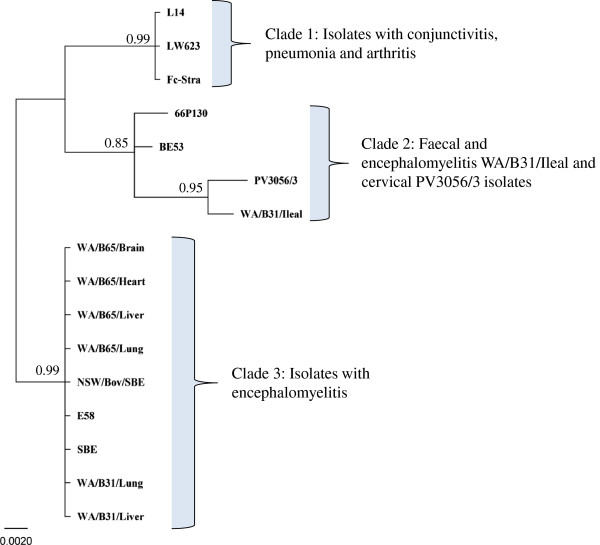
**Mid-point rooted Bayesian phylogenetic analysis of concatenated sequences of the seven HK gene fragments of the 16 bovine *C. pecorum *isolates used in this study.** Posterior probabilities of > 0.85 are displayed on the tree nodes.

Phylogenetic analysis revealed the presence of two genetically distinct *C. pecorum* STs in the samples analysed from the two animals (Figure 3). The predominant *C. pecorum* ST, detected in the brain, heart, lungs and liver from calves WA/B65 and WA/B31 was identical to the previously reported HK gene fragment sequences of the *C. pecorum* E58 SBE type strain, another SBE strain described in England and an Australian SBE strain (NSW/Bov/SBE), grouping them together in a well-supported Clade 3 (Figure 3). The second *C. pecorum* ST detected (WA/B31/Ileal) was found only in the ileal sample from calf WA/B31. This ST was clearly distinct from the other ST detected in these animals, differing by a total of 16 nucleotides, and clearly grouping in a well-supported clade together with other previously typed faecal (BE53 and 66P130) and cervical (PV3056/3) *C. pecorum* bovine genotypes (Figure 3). Phylogenetic analysis also revealed that both STs detected in these cases were also clearly distinct from a Clade 1 consisting of bovine isolates (Fc-Stra, L14 and LW623) previously associated with pathologies such as conjunctivitis, pneumonia and arthritis, respectively (Figure 3). MLSA of *C. pecorum* positive swab samples of healthy cohorts was not performed due to the low *C. pecorum* load. The phylogenetic distinction of the two *C. pecorum* genotypes observed in calf WA/B31, was supported by allele and ST identification with the ST from liver and lung determined as ST23, a ST previously described from Australian and the USA SBE isolates (NSW/Bov/SBE and E58), while the second *C. pecorum* ST from the ileal sample was assigned a novel ST since these sequences had not been previously reported (Additional file 1: Table S2).

We utilised pathological techniques to identify cases of SBE as well as a species-specific qPCR and a recently described *C. pecorum* MLSA scheme to molecularly characterise the strains of *C. pecorum* detected. This analysis revealed systemic dissemination of *C. pecorum* with associated pathologic changes. Molecular typing revealed the presence of two genetically distinct *C. pecorum* STs providing evidence for the presence of *C. pecorum* strains with different pathogenic potentials infecting cattle.

Histopathological findings were consistent with previous descriptions [2]. *Chlamydia*-specific IHC, targeting the genus-specific *Chlamydia*l polysaccharide (LPS), of affected tissues resulted in sporadic intense specific intracellular labelling of endothelium, intravascular mononuclear cells and mononuclear cells within inflammatory foci. Patchy intense labelling was seen in mononuclear cells (presumed macrophages or mesothelial cells) in superficial chronic exudative serosal lesions and individual mesothelial cells stained in less exudative lesions. These observations suggest that the initial site of infection in the brain is endothelium and in serosa, mesothelium. Detected antigen was not widespread in the tissues but appeared in clusters of cells. In one section of ileum (Figure 1A), rare individual cells within the intestinal epithelium and endothelial cells stained, suggesting a low level of infection in these sites. In the absence of *C. pecorum* specific antibody assays, we utilised highly sensitive *C. pecorum*-specific qPCR screening [18] to confirm these *Chlamydia*-specific IHC results in examined tissues. In contrast to these results, comparative screening by qPCR revealed a high average load of 10^4^*C. pecorum* 16S rDNA copies/μl of template in all tissues except the CSF sample. In several recent surveillance studies, PCR screening for *C. pecorum* has been shown to be an effective tool for assessing the overall herd prevalence from screening a limited selection of animals [29,30]. In Australian veterinary diagnostic laboratories, PCR testing for *Chlamydia* is not routinely performed but the results of this current study suggest that this should be reconsidered.

We also performed *C. pecorum* species-specific qPCR screening of asymptomatic in-contact animals. Nine out of 10 animals from the same mob were PCR positive for *C. pecorum* at ocular and vulval sites; however, the infectious load was very low (<15 *C. pecorum* 16S rDNA copies/μl of template). This observation is consistent with previous reports describing low levels of *C. pecorum* in vaginal swabs from cattle with no apparent disease [6,12,29]. The rectal swabs from in-contact animals in the current study were notably negative, raising questions about shedding, transmission and genetic relationships of strains particularly given the assumption that the gastrointestinal tract is the preferred site of infection for *Chlamydiae*[31]. Although unlikely, we cannot discount the presence of PCR inhibitors in these samples despite the fact that *C. pecorum* shedding in faecal samples of Australian cattle and sheep was previously observed and detected [15,32]. Unfortunately, molecular typing could not be performed on these swabs as the amount of *C. pecorum* positive DNA was insufficient for PCR amplification of the MLSA house-keeping genes. It has been hypothesised that subclinical infections may develop into diseases such as SBE by dissemination into other susceptible tissues via haematogenous spread [2], although the factors that determine this dissemination and disease development are unclear.

Previous observations that one animal can harbour two distinct *C. pecorum* genotypes from different anatomical sites [15], raised questions over the identity and genetic relationships between *C. pecorum* detected from multiple sites in both diseased and healthy animals in this study. To investigate this, we applied our *C. pecorum* MLSA scheme to reconstruct phylogenetic relationships [15] between the strains detected in the diseased calves and others previously described. This analysis revealed that Calf WA/31 harboured two distinct *C. pecorum* strain types, one in the gastrointestinal site (WA/B31/Ileal), apparently not associated with pathology and the other disseminated in multiple organs (WA/B31/Lung and Liver) and associated with severe pathology (Figure 3). These latter isolates had 100% sequence identity to other *C. pecorum* SBE isolates (E58, NSW/Bov/SBE and SBE), with a clear separation from isolates detected in bovine faeces. This observation of a phylogenetic distinction between SBE and faecal isolates has been previously described using an alternative multi-locus virulence typing scheme [16]. This is the first study to describe this observation in a single animal, however, further reinforcing that bacterial genetic differences may indeed be associated with differences in tissue tropism/pathogenicity [22]. As already mentioned, *C. pecorum* positive vaginal and ocular samples of healthy companion animals could not be typed due to very low levels of *C. pecorum*; however we hypothesise that if the MLSA was applied on these samples they would most likely cluster with the previously typed faecal isolates from healthy animals.

While the molecular evidence continues to mount for the existence of *C. pecorum* with different pathogenic potentials [22], this data needs to be supported by experimental infection studies. Rodolakis and colleagues in 1989, isolated intestinal *C. psittaci* strains (now reclassified as *C. abortus* and *C. pecorum*) from the faeces of healthy ruminants and showed in a mouse model that they were less “pathogenic” than strains isolated from animals with disease. Eight of 10 intestinal strains were non- invasive including the *C. pecorum* strains, while the majority of strains from diseased hosts, including *C. abortus*, successfully colonised the placenta and foetuses of pregnant mice [33]. To the best of our knowledge, more recent studies are currently lacking but will be required to experimentally verify the molecular evidence presented in this study. Molecular typing and experimental infection studies of the “true” pathogenic potential of *C. pecorum* will be particularly important in light of the growing number of studies which have linked *C. pecorum* infection to adverse but sub-clinical health consequences in dairy cattle [11,34].

## Conclusion

A combination of histopathological methods and molecular typing provided a new insight into the pathogenesis and epidemiology of SBE. Little is known about the prevalence and impact of *C. pecorum* infections on Australian livestock however this study provides a starting point for further investigations into cases of SBE in other regions of Australia and the relationship between *C. pecorum* infections and other health problems in Australian cattle. The detection of multiple genetically distinct *C. pecorum* strains that cluster with other disease associated or faecal strains reported in the rest of the world hint at a complex epidemiology in Australian animals that will require a combination of (i) large-scale ‘on-farm’ epidemiological studies; and (ii) further experimental infection studies with suspected “pathogenic” and “non-pathogenic” *C. pecorum* strains if we are to improve the diagnosis, treatment and control of these infections in livestock.

## Abbreviations

DAFWA: Department of Agriculture and Food, Western Australia; MLSA: Multi locus sequence analyses; PMN’s: Polymorphonuclear neutrophils; qPCR: Quantitative polymerase chain reaction; SBE: Sporadic bovine encephalomyelitis; ST: Sequence type.

## Competing interests

The authors declare that they have no competing interests.

## Authors’ contributions

MJ performed the molecular testing, data analyses and drafted the manuscript. DF performed the necropsy, histology and immunochemistry and assisted with preparation of the manuscript. JC and DR performed all field work. PT and AP conceived the study, assisted with data analysis and drafting of the manuscript. All authors read and approved the final manuscript.

## Supplementary Material

Additional file 1: Table S2 Allelic profiles of the 16 bovine *C. pecorum* used in this study. Click here for file

Additional file 2: Table S1 Sequence polymorphism analyses of the individual and concatenated 16 bovine *C. pecorum* HK gene fragments sequences used in this study. Click here for file
