# Supplementary material for: Molecular and pathological insights into Chlamydia pecorum-associated sporadic bovine encephalomyelitis (SBE) in Western Australia
Source: BMC Vet Res. 2014 May 29;10:121. doi: 10.1186/1746-6148-10-121 (PMC4064815; doi:10.1186/1746-6148-10-121)
Supplement: Additional file 1: Table S2 — Allelic profiles of the 16 bovine C. pecorum used in this study. [file 1746-6148-10-121-S1.pdf]

Table 1S. Sequence polymorphism analyses of the individual and concatenated 16 bovine *C. pecorum* HK gene fragments sequences used in this study

**Gene fragment**	**Sequence length (bp)**	**No. of non-synonymous substitutions**	**d_n^**	**No. of synonymous substitutions**	**d_s^**	**d_n/d_s**	**Δnt**	**N haplotypes**	**Haplotype diversity (H^x)**
*gat*A	425	2	0.00267	4	0.01724	0.15487	6	4	0.650
*opp*A_3	483	0	0	1	0.00117	0	1	2	0.125
*hfl*X	435	1	0.00071	3	0.00539	0.13173	4	5	0.667
*gid*A	474	1	0.00091	3	0.00818	0.11125	4	4	0.592
*eno*A	381	2	0.00261	8	0.02974	0.08776	10	6	0.675
*hem*N	432	0	0	1	0.00398	0	1	2	0.400
*fum*C	465	0	0	2	0.00466	0	2	2	0.233
Concatenated sequence	3095	6	0.00690	22	0.07036	0.09810	28	6	0.675

^d_s and d_n: the average number of synonymous substitutions per synonymous site and non- synonymous substitutions per non-synonymous site, respectively (Jukes - Cantor corrected); Δnt: No. of polymorphic sites; N haplotypes: No. of unique sequences according to the gene; H^x: measure of uniqueness of a particular haplotype in a given population (ranging from 0 (low haplotype diversity) to 1 (highly diverse)).
