# Supplementary material for: Molecular and pathological insights into Chlamydia pecorum-associated sporadic bovine encephalomyelitis (SBE) in Western Australia
Source: BMC Vet Res. 2014 May 29;10:121. doi: 10.1186/1746-6148-10-121 (PMC4064815; doi:10.1186/1746-6148-10-121)
Supplement: Additional file 2: Table S1 — Sequence polymorphism analyses of the individual and concatenated 16 bovine C. pecorum HK gene fragments sequences used in this study. [file 1746-6148-10-121-S2.pdf]

Table 2S. Allelic profiles of the 16 bovine *C. pecorum* used in this study

Isolate ID.	Host	Country	Pathology	ST	gatA	oppA	hflX	gidA	enoA	hemN	fumC
66P130	Bovine	USA	Subclinical (feces)	**49**	21	20	21	23	19	8	9
BE53	Bovine	England	Subclinical (feces)	**61**	21	12	26	23	20	8	20
E58	Bovine	USA	Encephalomyelitis	**23**	9	12	10	12	12	8	9
FC-Stra	Bovine	USA	Conjuctivitis	**48**	20	12	20	22	18	17	9
L14	Bovine	USA	Pneumonia	**48**	20	12	20	22	18	17	9
LW623	Bovine	USA	Arthritis	**48**	20	12	20	22	18	17	9
NSW/Bov/SBE	Bovine	AUS	Encephalomyelitis	**23**	9	12	10	12	12	8	9
SBE	Bovine	England	Encephalomyelitis	**23**	9	12	10	12	12	8	9
PV3056/3	Bovine	Italy	Metritis	**Novel ST**	31	12	10*	24	20***	17	20
WA/B31/Ileal	Bovine	AUS	SBE	**Novel ST**	21	12	10*	12	20****	8	20
WA/B31/Liver	Bovine	AUS	SBE	**23**	9	12	10	12	12	8	9
WA/B31/Lung	Bovine	AUS	SBE	**23**	9	12	10	12	12	8	9
WA/B65/Brain	Bovine	AUS	SBE	**23**	9	12	10	12	12	8	9
WA/B65/Heart	Bovine	AUS	SBE	**23**	9	12	10	12	12	8	9
WA/B65/Liver	Bovine	AUS	SBE	**23**	9	12	10	12	12	8	9
WA/B65/Lung	Bovine	AUS	SBE	**23**	9	12	10	12	12	8	9

*: denotes single nucleotide; ***: three nucleotides and ****: four nucleotides change to the reference allele.
